# Health Observation App for COVID-19 Symptom Tracking Integrated With Personal Health Records: Proof of Concept and Practical Use Study

**DOI:** 10.2196/19902

**Published:** 2020-07-06

**Authors:** Keiichi Yamamoto, Tsubasa Takahashi, Miwa Urasaki, Yoichi Nagayasu, Tomonari Shimamoto, Yukiko Tateyama, Keiichi Matsuzaki, Daisuke Kobayashi, Satoshi Kubo, Shigeyuki Mito, Tatsuya Abe, Hideo Matsuura, Taku Iwami

**Affiliations:** 1 Information Technology Center Wakayama Medical University Wakayama Japan; 2 beyondS Inc Tokyo Japan; 3 Wakayama City Public Health Center Wakayama City Japan; 4 Healthtech Laboratory, Inc Kyoto Japan; 5 Kyoto University Health Service Kyoto Japan; 6 Suzuka University of Medical Science Mie Japan; 7 TMI Associates Tokyo Japan; 8 Wakayama City Public Health Center Wakayama Japan

**Keywords:** public health informatics, public health administration, emerging infectious disease, preventive medicine, mobile apps, contact tracing

## Abstract

**Background:**

As a counter-cluster measure to prevent the spread of the infectious novel coronavirus disease (COVID-19), an efficient system for health observation outside the hospital is urgently required. Personal health records (PHRs) are suitable for the daily management of physical conditions. Importantly, there are no major differences between the items collected by daily health observation via PHR and the observation of items related to COVID-19. Until now, observations related to COVID-19 have been performed exclusively based on disease-specific items. Therefore, we hypothesize that PHRs would be suitable as a symptom-tracking tool for COVID-19. To this end, we integrated health observation items specific to COVID-19 with an existing PHR-based app.

**Objective:**

This study is conducted as a proof-of-concept study in a real-world setting to develop a PHR-based COVID-19 symptom-tracking app and to demonstrate the practical use of health observations for COVID-19 using a smartphone or tablet app integrated with PHRs.

**Methods:**

We applied the PHR-based health observation app within an active epidemiological investigation conducted by Wakayama City Public Health Center. At the public health center, a list is made of individuals who have been in close contact with known infected cases (health observers). Email addresses are used by the app when a health observer sends data to the public health center. Each health observer downloads the app and installs it on their smartphone. Self-observed health data are entered daily into the app. These data are then sent via the app by email at a designated time. Localized epidemiological officers can visualize the collected data using a spreadsheet macro and, thus, monitor the health condition of all health observers.

**Results:**

We used the app as part of an active epidemiological investigation executed at a public health center. During the investigation, 72 close contacts were discovered. Among them, 57 had adopted the use of the health observation app. Before the introduction of the app, all health observers would have been interviewed by telephone, a slow process that took four epidemiological officers more than 2 hours. After the introduction of the app, a single epidemiological officer can carry out health observations. The app was distributed for free beginning in early March, and by mid-May, it had been used by more than 20,280 users and 400 facilities and organizations across Japan. Currently, health observation of COVID-19 is socially recognized and has become one of the requirements for resuming social activities.

**Conclusions:**

Health observation by PHRs for the purpose of improving health management can also be effectively applied as a measure against large-scale infectious diseases. Individual habits of improving awareness of personal health and the use of PHRs for daily health management are powerful armaments against the rapid spread of infectious diseases. Ultimately, similar actions may help to prevent the spread of COVID-19.

## Introduction

The coronavirus disease (COVID-19) is widespread around the world. In clusters of the virus, there is likely to be a continuous generation of outbreaks, which may lead to larger-scale outbreaks [1]. In Japan, between 10% and 20% of all patients who are infected produce secondary infections [2]. Countermeasures aimed at preventing the spread of infection have centered on accurate detection and appropriate response to these clusters [3,4]. Accordingly, an effective and straightforward system for observing health status outside traditional health care settings would be useful as a counter-cluster measure for preventing the spread of COVID-19.

In Japan, COVID-19 is a designated infectious disease as defined by the relevant infectious disease control laws [3]. If a patient is suspected to have a COVID-19 infection based on clinical characteristics and diagnosis is confirmed by a polymerase chain reaction (PCR) test, the confirmed patient is hospitalized and isolated. An active epidemiological investigation is then conducted by public health centers, which are public institutions established by local governments based on the Japanese Community Health Act [5]. At public health centers, retrospective contact tracing [6-8] of confirmed patients and prospective symptom tracking [9,10] of their “close contacts,” people with whom they have been in recent contact, are conducted. First, to identify the source of infection and make lists of close contacts for the 14 days before the onset of symptoms, a retrospective behavioral investigation is conducted based on whether the confirmed patient had participated in events characterized by the “three Cs” (closed spaces with poor ventilation, crowded places with many people nearby, or close-contact settings such as close-range conversations) [11,12], as well as their travel history. Second, to prevent secondary infections, close contacts are monitored for fever, dyspnea, coughing, and other symptoms for 14 days from the most recent date of exposure to a confirmed patient. To relieve pressure on the medical system, further measures have been implemented to care for mild or asymptomatic patients at home or in isolated accommodations [13-15].

Health observations conducted outside of traditional hospital settings can be difficult. In one example, patients on a large cruise ship were confirmed to have COVID-19 [16-19]. After quarantine, the remainder of the passengers with negative PCR tests disembarked [20,21]. The Ministry of Health, Labor and Welfare (MHLW) issued a report on health follow-up, and the public health centers in the nearby residential area conducted a health observation [22]. Such an investigation is time-consuming because it is based on telephone interviews or similar formats [23]. In another example, a hospital doctor was confirmed to be infected, and 13 other individuals were confirmed to have been infected by that doctor within 10 days, mainly from close contact [24,25]. Such heavy investigation of close contacts prompted concerns that if the infection spread further, the public health center would be overloaded [23].

In Japan, which has become a super-aged society, it is critical to fill the gap between average life expectancy and healthy life expectancy [26]. Personal health records (PHRs) are expected to extend healthy life expectancy [27]. Based on their personal judgement, individuals record their medical, nursing, caregiving, and health-care–related data (ie, person-generated data [28]). Until now, this process has been considered suitable for daily management of an individual’s physical condition [29-32]. The PHR allows the user to check their own health status (eg, to be aware of their physical activity by measuring the daily number of steps or to prevent overeating by monitoring their daily weight measurements). Importantly for disease control, the PHR can be used to detect early signs of infection through regular measurements of body temperature. Management of an individual’s physical condition and self-care can theoretically be made easier by recording these data.

The Kyoto University Data Health Study Group, under the supervision of the Kyoto University Health Service, conducts research on shared lifelong PHR data based on annual health checkups for students and has created standardized models to promote the use of PHRs [33]. There are no major differences between the observation items collected by daily health observation for PHRs and the disease-specific observation items for COVID-19. We hypothesized that by expanding the PHR to collect observation items specific to COVID-19, it would be possible to efficiently observe the health of individuals outside of hospital settings, including the close contacts of confirmed infected people, as well as patients who are mildly symptomatic or asymptomatic at home or in isolated accommodations.

“K-note” (*Kenko-Nikki*; “health diary”) is a PHR-related app developed by Healthtech Laboratory, Inc, a Kyoto University–originated venture company in the Kyoto University Incubation Program [34]. The Kyoto University Data Health Study Group members and other volunteers, including researchers at Wakayama Medical University, gathered data and added health observation functions specific for COVID-19 to the PHR data.

The objective of this study is to determine whether PHRs could be used for efficient health observation of emerging infectious diseases among individuals outside a traditional hospital setting. In addition, we sought to demonstrate the practical use of a smartphone and tablet app that supports PHR-based health observation by integrating monitoring functions specific to COVID-19. We explored the development, use, and efficiency of this app relative to conventional methods in the setting of an actual active epidemiological investigation of COVID-19.

## Methods

### Basic Concept and Features of K-Note

The PHR smartphone and tablet app “K-note” was developed to manage various data based on an individual’s input as an alternative to conventional paper-based health checkup information. It is used to record day-to-day health information such as the number of steps taken, body weight, and blood pressure, as well as medical information such as medications and vaccination history. Its principal purpose is to improve individual lifestyles and, ultimately, extend healthy life expectancy.

Since July 2019, K-note has been distributed free of charge to study participants as part of a proof-of-concept study of health promotion using PHRs. We recently added a COVID-19 health observation function to the PHR app and made the software available to the public free of charge.

Initially, the integration of health observation data specific to COVID-19 in the K-note app was intended to streamline health observation within schools and companies of groups suspected of being infected. However, following an incidence of COVID-19 infection, at the request of Wakayama City Public Health Center, the app was applied in the context of an active epidemiological investigation [24]. To improve the efficiency of health observation work at the public health center, we referred to the survey items of the National Institute of Infectious Diseases [35] and created a Microsoft Excel (Microsoft Corporation) macro for data visualization.

In general, personal information protection and network security in Japan is strict, and there are often severe restrictions on access to webpages and social networking services, as well as personal computer settings. Because K-note is intended to be used for health observation related to COVID-19, it was designed to be usable in various information and communication technology environments, including use by local governments. The basic concepts underlying development are as follows: only the app and the readily available Excel software package are required, operation can start immediately, and due consideration is given to the protection of personal information. The benefits of the app include its availability as a free download (ie, App Store, Google Play). Data specific to COVID-19 can be recorded and managed with other health observation data, and can be sent by email to the specified destination with one click. Individuals can easily review their own health observation data, and the epidemiological officer can easily visualize the comma-separated values (CSV) data they have received using the Excel macro for data visualization. Data are managed only within the smartphone or tablet and are not made available elsewhere unless the individual who has been in close contact with an infected individual and is to be observed (health observer) specifically authorizes data sharing. K-note uses the health observation form to show the state of the health observer as “not infected” (ordinary health follow-up, close contact, etc) or “diagnosed with infection” (mild or asymptomatic), and users can choose from these options. Here, we show an example of ”not infected“ (ordinary health follow-up, a close contact of a known infected individual).

### Overview of Health Observation for Close Contacts at the Public Health Center Using K-Note

**Figure 1:**
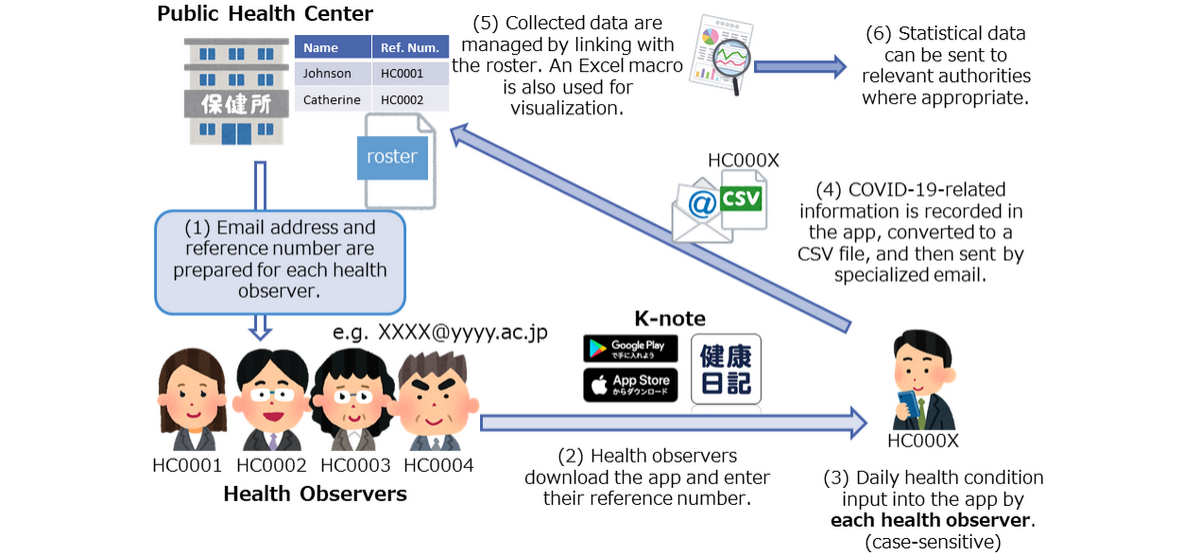
Overview of health observation of close contacts at the public health center using K-note. CSV: comma-separated values.

At the public health center, a list of health observers is made; a reference number, used in management, is assigned at this time. A special email address is also created that is used when the health observer sends data to the public health center. Every day by the designated time, the health observer uses the integrated email address to send their data related to COVID-19, such as body temperature and symptoms, which were recorded by the app. When the information has been received by the epidemiological officer at the public health center, they can visualize the collected CSV data using an Excel macro, and this can be used to monitor the health condition of all health observers. If appropriate, statistical data can be sent to authorities such as the MHLW and quarantine stations (Figure 1).

Health observation at the public health center using K-note is performed in three phases: preparation, health observation by K-note, and data visualization at the public health center.

### Phase 1: Preparation

As part of an active epidemiological investigation, the condition of confirmed patients is investigated at the public health center, and a list of health observers, who are to be observed for 14 days, is made. The epidemiological officer at the public health center acquires a reference number for each health observer. The ”roster“ sheet of the Excel macro for data visualization serves as the epidemiological officer management’s ledger of health observers.

**Figure 2:**
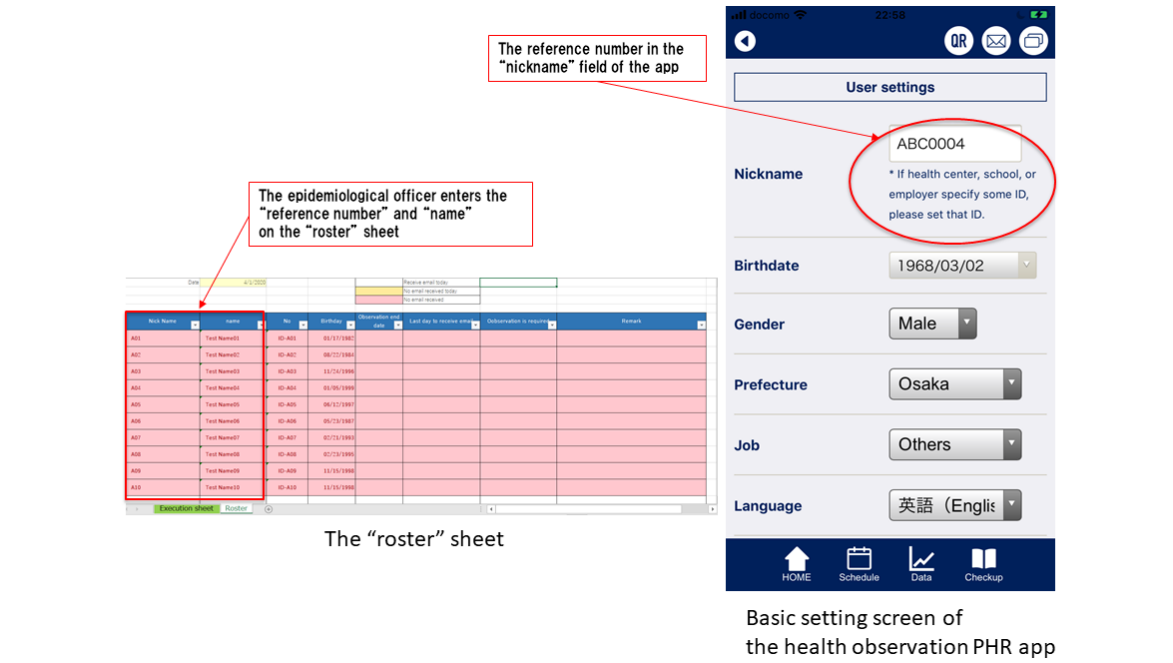
The “roster” sheet and the basic setting screen of K-note. PHR: personal health record.

Because unique email addresses are used to transmit data to the public health center, the epidemiological officer can monitor each health observer separately, which may facilitate the investigation. Although this is the system we have used, it may be useful to create the email address differently according to the operations of the public health center (eg, by creating one for each epidemiological officer at the public health center or one for each group of close contacts; Figure 2).

### Phase 2: Health Observation Using K-Note

**Figure 3:**
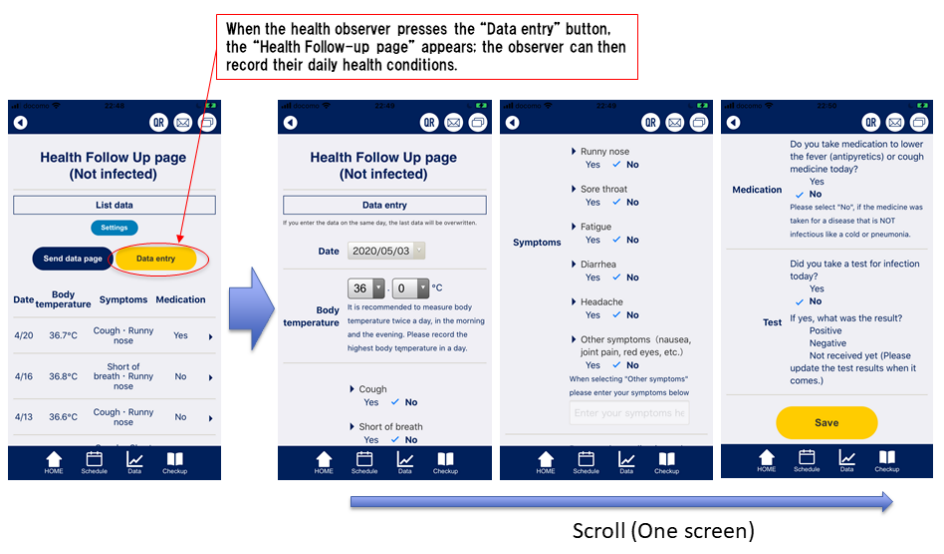
Health observation data input screen in K-note. Data entry items are body temperature, coughing, dyspnea, rhinorrhea, sore throat, fatigue, diarrhea, headache, other symptoms, medication, confirmation of whether a test has been taken, and test results.

The health observer records their daily health conditions such as body temperature and symptoms for 14 days from the start of the observation (Figure 3).

**Figure 4:**
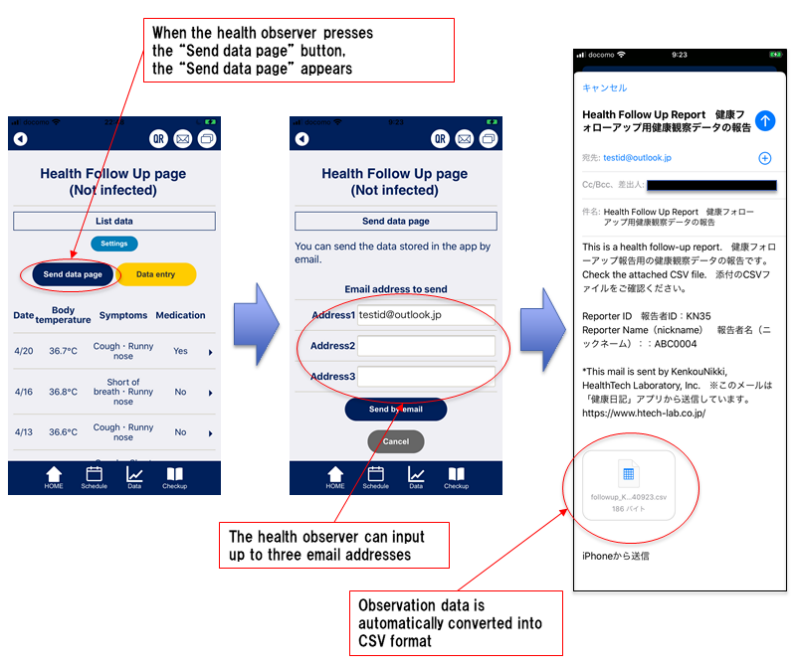
Email sending screens. CSV: comma-separated values.

**Figure 5:**
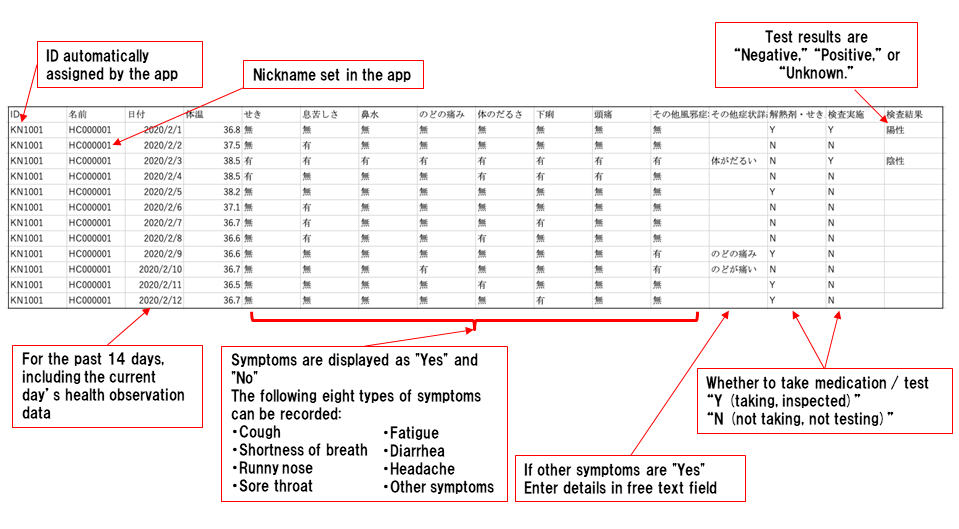
Transmitted comma-separated values data image.

After data input, when the health observer presses the “send data page” button, the “send data page” appears. Health observers may input up to three email addresses. When the health observer presses the “send by email” button, the observation data is automatically converted into CSV format and attached to the email. By a designated time every day, the app is used to send the health observer’s recorded observation data to the specified email addresses (Figures 4 and 5).

### Phase 3: Data Collection by the Epidemiological Officer at the Public Health Center

**Figure 6:**
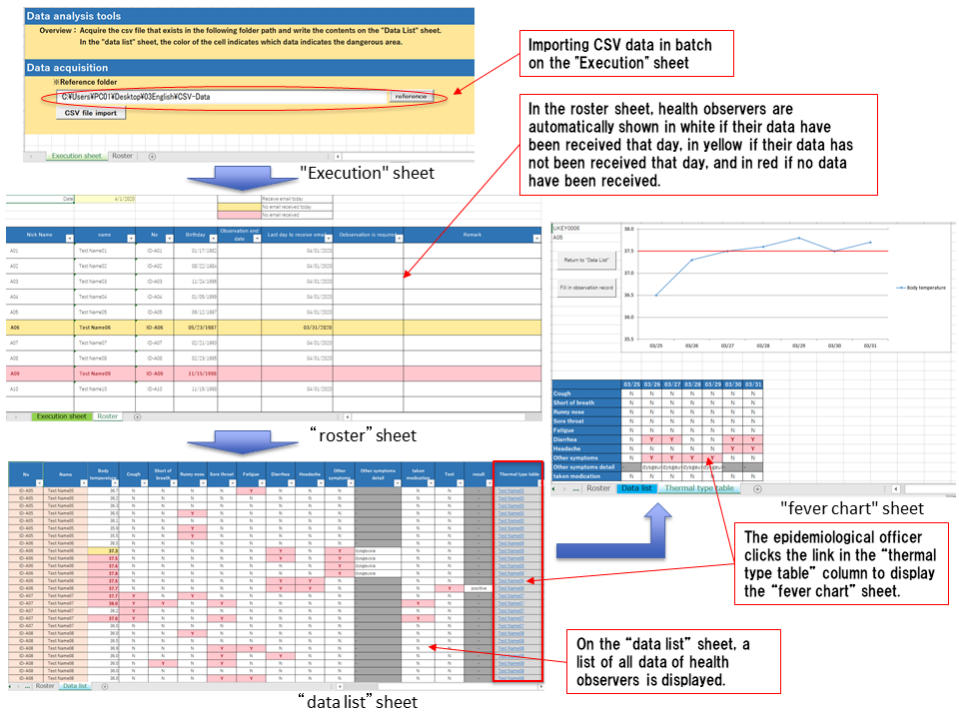
Screen images of the “execution,” “roster,” “data list,” and “fever chart” sheets. CSV: comma-separated values.

The epidemiological officer downloads the CSV data of all health observers received on that day and stores them in a single folder. By launching the Excel macro for data aggregation and batch importing the CSV data on the ”Execution“ sheet, all health observation data received on that day will be displayed on the ”data list“ sheet, and the “roster” sheet is updated. In the ”roster“ sheet, health observers whose health observation data were received that day are displayed in white, those whose data was not received on the day are displayed in yellow, and those whose data has never been received are automatically displayed in red. With this function, the epidemiological officer can easily distinguish the reporting status of all health observers. It is possible to adapt this system according to the situation (eg, it may be sufficient to contact only the health observers who are displayed in yellow or red). On the ”data list“ sheet, all the data for all the health observers are displayed. Excel functions can be used to rearrange the health observers and display only a particular day. Regarding body temperature, yellow is automatically used to distinguish health observers whose reported body temperature was ≥37°C, and red is used to distinguish those with temperatures ≥37.5°C. Coughs or other symptoms are automatically displayed in red. If the epidemiological officer checks the “data list” sheet and a health observer requires more detailed observation, they may click the link in the ”Thermal type table“ column to display the ”fever chart“ sheet. Here, body temperature is displayed graphically over time, and 37.5°C is indicated by a red line so that it is possible to see at a glance whether there are days when the temperature exceeded the standard value. Symptoms are also listed over time and displayed in red (Figure 6).

## Results

### Active Epidemiological Investigation Executed by the Public Health Center

We started developing the health observation app on February 22, 2020, and registered the initial version in the app store on February 27. On March 5, a patient with COVID-19 at a workplace in Wakayama City was confirmed, and the use of the initial version of the app was started at the Wakayama City Public Health Center on March 6. We immediately customized the health observation app according to the actual epidemiological investigation and released an updated version on March 15. Health observation had been performed for 14 days starting from the day of contact (exposure) with the confirmed patient. In principle, the investigation would be tracked daily until all health observers were contacted. For those who were exposed before March 5 and were found to be infected by health observation, observation will be completed without waiting for the final day. During the active epidemiological investigation, the Wakayama City Public Health Center discovered 72 health observers who had close contact with a confirmed case. Among them, 57 (79.2%) adopted the use of the health observation app and 14 used telephone investigations; the observation period of the remaining health observers ended on March 5 (Table 1).

**Table 1:** Results of health observation from March 6, 2020.

Date in 2020		Day of week	Investigation using the app		Investigation by phone	
			Health observers, n	Spontaneous email senders, n	Health observers, n	Those who could be contacted by one phone call, n
**Total**			729	632	171	154
	March 6	Friday	57	48	14	14
	March 7	Saturday	57	54	14	14
	March 8	Sunday	57	56	14	13
	March 9	Monday	57	54	14	14
	March 10	Tuesday	57	49	14	13
	March 11	Wednesday	56	44	14	14
	March 12	Thursday	53	45	12	8
	March 13	Friday	53	45	11	8
	March 14	Saturday	51	44	11	10
	March 15	Sunday	50	39	11	10
	March 16	Monday	50	44	11	11
	March 17	Tuesday	49	40	11	11
	March 18	Wednesday	46	40	10	7
	March 19	Thursday	36	30	10	7

During the app-based investigation, the 6 health observers who did not send an email on March 19, 2020, were contacted by phone by an epidemiological officer to inform them of the end of the observation; their health condition was checked at that time. In total 57 of 72 health observers (79.2%) chose the app. The spontaneous email transmission rate was 86.7% (n=632/729).

Before the app was introduced, all health observers were interviewed by telephone, a process that took four epidemiological officers more than 2 hours. This suggested that, should the virus spread further, it would be difficult to maintain investigations by telephone with limited staff over 14 days. Additionally, the comprehensiveness of the investigation may be compromised because health observers might be absent or epidemiological officers might not be able to make a phone call because of other work. Furthermore, the health observer also had to wait in a place where they could answer the telephone during the survey period, placing a heavy burden on both the health observer and the epidemiological officers. With the introduction of K-note, it became possible to automatically manage information collectively; the health observers sent their data at a designated time every day, and a single epidemiological officer could carry out health observations alone. In addition, the arrival status of observation data and the health condition of the health observer were readily visualized, which improved the efficiency and comprehensiveness of the investigation. To improve efficiency, the app was modified 6 times, and the Excel macro 3 times, by mid-May.

### Usability Evaluation of K-Note

**Figure 7:**
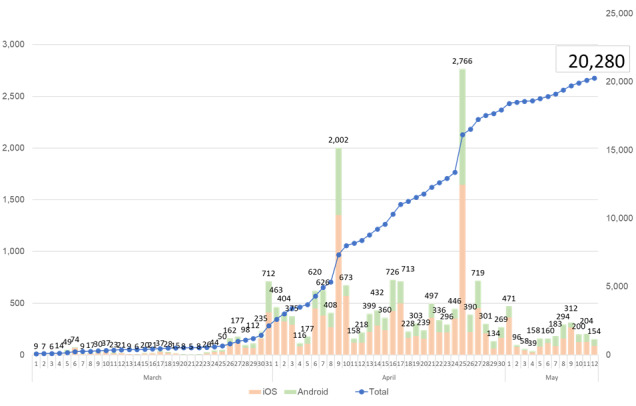
The number of app users between March 1 and May 12, 2020.

**Figure 8:**
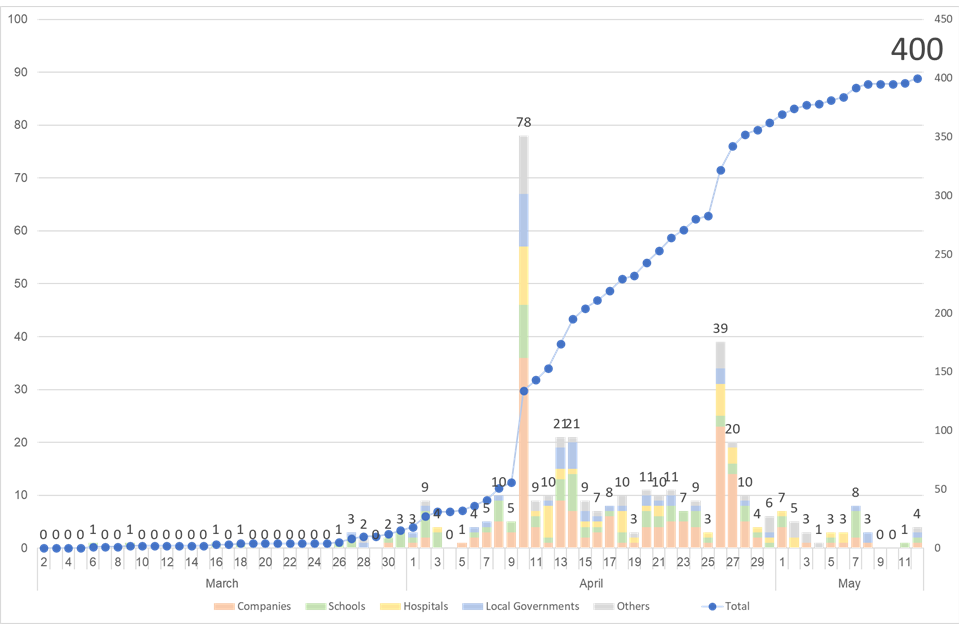
The number of facilities and organizations between March 1 and May 12, 2020.

As of May 12, 2020, K-note has been used by more than 20,280 individuals and 400 facilities or organizations, centered on companies, schools, hospitals, and local governments in Japan. The breakdown by facility was as follows: 166 (41.5%) companies, 90 (22.6%) schools, 51 (12.7%) hospitals, and 47 (11.7%) from local government. In addition, it has been introduced in 24 of 47 prefectures and is used in 35 public health centers (Figures 7 and 8).

At the time the app was released, only email support was implemented, but with the increase in the number of inquiries, a help desk officially opened on March 28. By mid-May, the total number of inquiries to the help desk was 99. The breakdown of inquiries was: 18 (18%) “Can’t send emails from the app,” 17 (17%) “App functions and usage,” 20 (20%) “Can’t download Excel macro,” 26 (26%) “Excel macro function and usage,” and 18 (18%) “Other inquiries.” In addition, online operation briefing sessions for the app were held eight times by mid-May for a total of 152 participants.

To evaluate the usability of the app, we interviewed the help desk manager about the context of use, effectiveness, efficiency, and satisfaction according to the definition of usability in ISO 9241-11:2018 [36]. The results are shown in the following sections.

#### Context of Use

The use of apps started with local governments and gradually spread to schools and companies. Initially, a tool for health observation was required as a COVID-19 countermeasure, and many facilities decided to introduce our app because of the motivation to respond promptly rather than seeking efficiency and effectiveness. Many users expressed their gratitude for an Excel-based system that could be introduced immediately, even if it was a bit inconvenient, because they did not know the specific method for health observation and there was no alternative system. As social awareness of the need for health observation increased, the number of users from large universities and companies increased. Since mid-May, the number of inquiries by health observers has increased due to the state of emergency being lifted in Japan and the subsequent movement toward resuming social activities [37].

#### Effectiveness

From the viewpoint of effectiveness, the ability to record daily health condition was of great significance as a countermeasure against COVID-19. Because it was possible to look back when some problem occurred, the app could be used for health observation not only in active epidemiological investigation at public health centers but also for health observation before and after events such as educational practice, face-to-face lectures, and physical education classes.

#### Efficiency

Because of the rapid spread of COVID-19, no one was demanding optimal efficiency. Still, we must acknowledge that our method for exchange of CSV data by email and manual data visualization by Excel macro was not efficient. In addition, there were many inquiries from local governments and companies indicating that, for security reasons, it was not possible to use web forms for the online briefing sessions or to download files (including the Excel) macro on the internet.

In the flood of various information technologies, the following two points are presumed to have driven increased demand for our app.

1. Among members of the general public, there was not a habit of taking daily measurements of body temperature and monitoring changes over time to lead to behavioral changes without a doctor's guidance; however, there was no alternative system that could also manage personal consent and security.
2. Even the local government had no experience with exchanging health information electronically with the general public.

#### Satisfaction

From the viewpoint of satisfaction, the administrator of a facility or organization was able to fulfill their social responsibility by introducing our app as the infection of COVID-19 spread. Health observers also had a sense of security that someone was observing their health condition. Therefore, it could be presumed that satisfaction was high. Recently, health observation became one of the requirements for resuming social activities in Japan.

## Discussion

### Principal Findings

We extended PHRs and developed a PHR-based health observation app for counter- cluster measures of COVID-19. As a result of applying the new app in an active epidemiological investigation at a public health center, we believe that the efficiency and completeness of the investigation have been improved, preventing the spread of infection. The app is used nationwide in Japan, mainly by companies, schools, hospitals, and local governments for health observation for the prevention of COVID-19. Except for active epidemiological investigations at public health centers, confirmed patients may not have been identified by self-reporting from schools or companies alone. However, the app has contributed to the early detection of COVID-19 or voluntary self-quarantine at home by individuals with suspected symptoms, and the use of the app can facilitate reopening of school and corporate activities. Our findings suggest that health monitoring of PHRs is useful for carrying out efficient health observation of emerging infectious diseases for individuals outside of a traditional hospital setting.

A factor contributing to the success of our app is that we assumed that it would be used for health observation of small groups; accordingly, we designed the system configuration so that it could be introduced immediately without a cloud system; the user only requires the app itself and Excel. Many local governments in Japan have strict control of security and protection of personal information, and are often restricted from displaying external webpages and using social networking services from their office local area networks. A system for storing health observation data in the cloud and sharing data between the health observer and the public health center would require security-related deliberation and entail large-scale initiatives for entire cities or prefectures. On the other hand, our method can be implemented immediately and at low cost in a comparatively simple ICT environment in which emails can be exchanged between the health observer and the public health center using just the app and Excel. It is therefore possible for each public health center to make a decision about whether to introduce the system. Additionally, because it was based on the COVID-19 health observation items of the National Institute of Infectious Diseases and reflected the opinions of preventive medicine specialists and epidemiological officers at public health centers, the app was practical.

The basis of this study was the idea that PHRs, which are used to promote health via daily observation, can be used as a counter-cluster measure against large-scale infectious diseases. In the case of highly infectious diseases, infection is often found in people in close contact with a known infected patient, but they often do not have symptoms at the start of the observation. For this reason, it is extremely difficult to undertake comprehensive health observations outside the hospital, including of asymptomatic individuals or those with mild symptoms, those under isolation, those who are suspected to be infected based on airport quarantine, and those within active epidemiologic investigations. It is obviously too late to start preparations after an infectious disease begins to spread, and ideally, a system would be established during normal times. However, it is socially difficult to invest in and maintain costly measures against large-scale infectious diseases that occur once every few years or even several decades. Although there are observation items specific to infectious disease control, they are just an extension of daily health observation. We believe that the idea of converting daily health promotion activities by PHR into countermeasures against large-scale infectious diseases in emergencies could enable a swift response to these illnesses.

The popularization of PHRs is key to making daily health observations the basis of counter-cluster measures against large-scale infections. It is important for promoting the significance of the habit of self-management of health data and as a tool for individuals to maintain their own health throughout society. As a countermeasure against COVID-19, many people have undertaken health observation using our app in Japan. We would like to further promote the habit of PHR-mediated health observation as a social infrastructure even after the end of COVID-19. To do that, more people must become aware of the benefits of maintaining PHRs. Taking advantage of the experience of developing a health observation app for COVID-19, we would like to develop various additional apps of the same kind, not only as a measure against emerging infectious diseases but also for health management for public marathons, in club activities in educational institutions, and after large-scale disasters.

### Limitations

For research, detailed quantitative and qualitative evaluation items should be determined, and data should be collected at the time that the research plan is created. However, due to the urgency of preventing the spread of COVID-19, app development and an introduction to public health centers were given priority. In this case, we only performed qualitative evaluation of the burden reduction effect by interviewing epidemiological officers and help desk managers. Another limitation is that some members of society do not have access to smartphones, tablets, or Wi-Fi connections. Although the process is explained well here, some people may find the method difficult to use if they are not familiar with information and communications technology. We distribute to these health observers an Excel survey sheet that can be read into the data visualization Excel macro. In addition, a proxy input function may be necessary. The current Excel macro for data visualization was developed for the health observation of individuals who have been in close contact with known infected patients at public health centers. We believe that this is suitable for surveys of up to 100 close contacts per confirmed patient. If the number of people exceeds 100, a more sophisticated data visualization system may be required. It would also be possible to develop a retrospective behavioral investigation support app for confirmed patients and close contacts using smartphones and PHR behavioral history information, but such an app has not been developed due to concerns about protecting personal information [38,39]. Currently, contact-tracing apps are being developed all over the world [6-8]. In Japan, as a counter-cluster measure, retrospective behavioral investigation of confirmed patients, explorations of close contacts, and prospective symptom tracking of close contacts are executed as active epidemiological investigations, but the amount of work required is enormous. If contact-tracing and symptom-tracking apps [9,10,40] can be used in combination in an active epidemiological investigation, it may be possible to greatly reduce the workload.

As a measure against future large-scale infectious diseases after the termination of COVID-19, it would be useful to develop an infectious disease control support network based on PHRs that could seamlessly respond to signs of infectious disease spread active case findings of behavioral investigation of confirmed patients and close contacts, health observation of out-of-hospital close contacts and mild or asymptomatic patients, and provide statistics to local governments and national headquarters.

### Conclusions

We developed a health observation app integrated with PHRs as a COVID-19 counter-cluster measure. The app greatly reduced the follow-up burden of individuals who had close contact with known cases of confirmed COVID-19 infection. The relatively low-tech nature of the app and Excel combination meant that it was easily accessible, especially in Japan. The system uses emails rather than the cloud, an approach that is arguably more compatible with business and privacy practices specific to Japan. Health observation by PHRs for the purpose of improving health management and extending healthy life expectancy by using individuals’ own health information is also effective as a countermeasure against large-scale infectious diseases. We believe that raising the awareness of each person about their own health and the value of using PHRs for daily health management is a powerful weapon against the rapidly expanding spread of infectious diseases. We hope that our study will help prevent the spread of COVID-19 infections and future large-scale infectious diseases around the world.

## Appendix

Multimedia Appendix 1 Excel macro and demo data (demo_data_visualization.zip).

## Abbreviations

COVID-19: coronavirus disease
CSV: comma-separated values
MHLW: Ministry of Health, Labor and Welfare
PCR: polymerase chain reaction
PHR: personal health record
